# Motor Outcome After Posterior Insular Resection for Pediatric Epilepsy

**DOI:** 10.3390/brainsci15020177

**Published:** 2025-02-11

**Authors:** Michael E. Baumgartner, Samuel B. Tomlinson, Kathleen Galligan, Benjamin C. Kennedy

**Affiliations:** 1 The Perelman School of Medicine at the University of Pennsylvania, Philadelphia, PA 19104, USA; michael.baumgartner@pennmedicine.upenn.edu; 2 Department of Neurosurgery, University of Pennsylvania, Philadelphia, PA 19104, USA; samuel.tomlinson@pennmedicine.upenn.edu; 3 Division of Neurosurgery, The Children’s Hospital of Philadelphia, Philadelphia, PA 19104, USA; galligank@chop.edu

**Keywords:** insula, epilepsy, insulectomy, surgery

## Abstract

The increasingly widespread use of stereo-EEG in the pre-surgical evaluation has led to greater recognition of the insula as both a source and surgical target for drug-resistant epilepsy. Clinicians have long appreciated the challenges of diagnosing and treating seizures arising from the insula. Insular-onset seizures present with a wide variety of semiologies due to its dense and complex integration with other brain structures, resulting in the insula’s reputation as the “great mimicker.” Surgical access to the insula is guarded by the overlying frontal, temporal, and parietal opercula and requires careful negotiation of the Sylvian fissure, the vascular candelabra of the middle cerebral artery, and protection of crucial white matter structures (e.g., corona radiata). Despite these difficulties, open surgical intervention for insular epilepsy is associated with favorable seizure control rates, surpassing those achieved with less-invasive alternatives (e.g., laser ablation). Technical nuances that minimize the risk of adverse functional outcomes following open insular resection (especially hemiparesis) are of tremendous value to the epilepsy surgeon. Here, we review the literature on hemiparesis secondary to insular resection and detail strategies for achieving safe and thorough resection of the insula, with emphasis placed on the posterior insula. We supplement this review with four illustrative cases in which focal, drug-resistant epilepsy was managed via open insular resection with no resultant permanent hemiparesis. Technical insights accumulated through these cases are highlighted.

## 1. Introduction

Focal drug-resistant epilepsy arising from the insula is challenging to diagnose and difficult to manage. Historically, detection of insular-onset seizures has been limited by its deep anatomic location and inaccessibility with scalp or surface neocortical electrodes [1]. sEEG has made insular recordings practicable and enabled a clearer understanding of the prevalence and semiology of insular-onset seizures, as well as generating increased interest in surgical management [2,3,4]. The challenge of diagnosing insular epilepsies is compounded by semiology: the rich, complex integration of the insula with surrounding brain structures results in seizures that can resemble those of frontal, temporal, and parietal origin [1,3]. Accurate localization of insular seizures is further complicated by the fact that these seizures may exhibit rapid propagation to the contralateral insula [3]. Insular seizures have been associated with semiologies as diverse as autonomic, hypermotor, viscerosensory, olfactory, gustatory, auditory, and language disturbances, though insular seizures typically begin with preserved awareness [3]. This is consistent with the insula’s poorly understood but diverse functional anatomy [3]. One semiology highly associated with insular seizures, first identified by Isnard et al., consists of laryngeal constriction, cutaneous paresthesias, and preserved awareness at seizure onset, followed by dysarthria and focal motor symptoms [1,5,6].

Open surgical resection for insular-onset epilepsy is recognized as a technically challenging procedure with associated risks of debilitating morbidities [7]. Hemiparesis secondary to manipulation of the MCA candelabra and infarct of the corona radiata is a feared and relatively common complication of insular resection, with reported rates of contralateral hemiparesis as high as 40% in previous series [1,5,7,8]. The difficulty of open insular resection has resulted in the increased popularity of minimally invasive alternatives, such as laser interstitial thermal therapy (LiTT), which are proposed to offer a safer approach for disrupting insular seizure foci [2]. There are retrospective data in the literature that suggest that open resection achieves superior rates of seizure freedom. A recent meta-analysis by Obaid et al. suggests that seizure outcomes following stereotactic ablation (laser or radiofrequency) are inferior to those achieved via open resection [7]. A study by Hale et al. found a higher rate of Engel class I outcomes for patients undergoing open resection versus LiTT; however, the difference was relatively small (50% vs. 43%), and they concluded that both approaches are valid management options [9].

Performing safe and efficacious surgery for insular epilepsy demands a thorough understanding of insular anatomy and an appreciation of technical nuances that protect the surgeon during key steps of the operation. In this article, we present our recent experience with insular resections at the Children’s Hospital of Philadelphia. We focus on the approach to the posterior insula, which overlies the posterior limb of the internal capsule and permits passage of arterial perforators supplying the corona radiata. We review motor outcomes from these cases and highlight key technical considerations for avoiding motor deficits while performing posterior insulectomies.

### 1.1. Anatomic Considerations

The insula consists of a thin rind of gray matter, deep to which are the extreme capsule, claustrum, external capsule, lentiform nuclei, and internal capsule [10]. The insula is situated deep to both the Sylvian fissure and the opercula of the frontal, temporal, and parietal lobes. In the dominant hemisphere, the posterior insula sits deep to canonical Wernicke’s area, and the arcuate fasciculus runs superior and posterior to the circular sulcus at the posterosuperior margin of the insula [11,12]. Optic radiations may also be at risk during surgery [12]. The anterior insula typically consists of three short gyri, as well as the accessory and transverse insular gyri, and is bounded anteromedially by the limen insulae. The posterior insula consists of the anterior and posterior long gyri [11,13]. The anterior and posterior insulae are separated by the central sulcus of the insula. The genu of the internal capsule is deep to the middle third of the insula, and the posterior limb of the internal capsule and thalamus are deep to the posterior insula [13]. M2 and M3 MCA branches pass directly over the insular surface, with arteries running in all insular sulci except the superior limb of the circular sulcus [13]. The blood supply to the insula derives predominantly from small branches off the overlying M2 vessels. Some long insular arteries penetrate the insula to supply deep structures, including the corona radiata, typically in the superior portion of the posterior long gyrus [13]. This blood supply appears to be relatively uncommon, only occurring in 3–5% [14] and 11% [13] of hemispheres in cadaveric studies. These anatomical studies, however, may underestimate the frequency of this blood supply, as infarcts in the corona radiata are common following insulectomy, resulting in typically transient hemiparesis [8]. Another possibility is that corona radiata infarcts following insulectomies could often result from damage to medullary arteries arising from opercular and cortical MCA segments, which have been found to supply the corona radiata in cadaveric microangiography [7,15]. The lateral lenticulostriate arteries are anatomically far from the posterior insula but may be vulnerable when operating anteriorly near the limen insulae, which lies directly lateral to the anterior perforated substance, and perforators can emerge as far lateral as the MCA bifurcation [13]. The posterior insula is, therefore, bounded by critical cortical, white matter, and vascular structures, which place motor and language function at risk during surgery.

### 1.2. Reaching the Posterior Insula

The posterior insula can be approached either by trans-sylvian or trans-opercular approaches. Examples of both strategies are detailed below. In general, the trans-sylvian corridor allows for resection of the insula without damage to overlying structures. This comes, however, with the risk of venous infarct due to damage of the cortical veins overlying the fissure, as well as cortical, corona radiata, and basal ganglia strokes resulting from manipulation or damage of the fissural MCA branches. There is a further risk of retraction injury to the operculum. The trans-sylvian approach provides excellent exposure of anterior and inferior aspects of the insula down to the limen insulae [13]. The superior and posterior aspects of the insula are more challenging to reach via this approach and may necessitate excessive opercular retraction as the thickness of the operculum increases posteriorly [13]. These risks can be reduced in surgical cases with peri-sylvian injury or any pathology that widens the Sylvian fissure and expands the natural surgical corridor. Bouthellier et al. advise against approaching the insula at an angle directly perpendicular to its surface, instead advocating for a tangential angle of approach to enable a greater extent of subpial resection16. Patient positioning should be conscious of this, and they advocate that the head be positioned horizontally with a downward tilt when performing an insulectomy following a temporal operculectomy or via a trans-sylvian approach, head up when following a frontal or parietal operculectomy, and neutral for radical operculectomy [16]. If greater exposure of the insula is required, they advocate opening the chiasmatic cistern for CSF drainage and brain relaxation [16]. In general, the trans-sylvian approach is suitable for insular lesions without opercular involvement, as long as care is taken to widely open the fissure [12]. The Sylvian fissure should be opened widely to ensure adequate visualization of the area to be resected to minimize retraction and reduce the risk of under-resection [12].

A trans-opercular approach, by contrast, offers the advantage of wider exposure, and the pia overlying the operculum provides a further layer of protection against injury to vascular structures in the Sylvian cistern. Such an approach, however, comes at the expense of greater cortical disruption and, in the dominant hemisphere, risks damage to critical language structures. In the case of lesions involving both the insula and the overlying operculum, resection of the lesion itself can create adequate surgical exposure of the deeper insular structures. For trans-opercular insulectomy, Bouthellier et al. recommend removing two centimeters of operculum away from the Sylvian fissure so as to enable a tangential angle of approach to the insula [16].

Contraindications for insular resection are similar to those for all resective epilepsy surgeries, the most important of which is the inability to clearly identify the seizure onset zone, which can be particularly challenging for the insula. As many patients with epilepsy, due to age or disability, are unable to participate in formal language testing or awake craniotomy, particular care must be paid to the risks of language deficit and whether the surgery can be safely performed, given the proximity to the language cortex. Furthermore, conditions that severely distort the anatomy of the insula and peri-insular region warrant further planning as to whether or not the surgery can be safely performed.

Another consideration is the intraoperative assessment of larger perforating arteries of the posterior insula. Under the operating microscope, gentle manipulation of M2 vessels can sometimes reveal where larger perforating arteries arise. Once identified, these locations can serve as areas to work around. There are two approaches to resection of any given part of the cortex: through the insular pia and under the insular pia. In any insular resection, a combination of both is typically used. When part of the operculum has been resected, this often allows for more resection under the pia, but for a large resection, even with operculectomy, often the best way to avoid disrupting all perforating arteries is to perform some of the insular resection, often farthest from the opercular resection, with one or two new transpial openings through the crowns of insular gyri.

### 1.3. Preservation of Function

Preservation of function in this surgery is contingent upon robust surgical technique applicable to any corticectomy near to eloquent cortex, in addition to these specific anatomic considerations. Monopolar stimulation is recommended for mapping the motor and sensory strips if exposed during the course of the surgery. Preoperative language mapping is recommended for patients of sufficient age and maturity to participate. While it is rare for a pediatric epilepsy patient to be able to tolerate and effectively participate in an awake craniotomy, such an approach is advisable when operating in the frontoparietal operculum and anterior insula of the dominant hemisphere, if possible. Focal epilepsy can be cured by adequate gray matter resection while sparing white matter [17]. Given the thin insular cortex and vulnerable white matter structures at risk, we recommend resection of gray matter using suction or, in cases of gliosis, ultrasonic aspiration at minimum necessary settings using careful subpial technique. Bouthellier et al. recommend using suction against the pial surface in order to prevent plunging into the underlying putamen [16]. With the exception of the posterosuperior insula, small arteriolar branches entering the insula can be sacrificed during the skeletonization of overlying vessels without risk of functional deficit, as these arteries supply the insular cortex being resected. Preservation of sulcal and pial anatomy is critical for maintaining surgical orientation, and the resection margins should respect sulcal anatomy with dissection down to and just under the depths of the sulcus, following the gray–white junction visually. White matter should be minimally violated, and there is no benefit to chasing gliotic or radiographically abnormal white matter deep into the insula, which would also increase the risk to deep structures and blood vessels. Splitting the fissure widely or performing a complete insulectomy necessitates operating in the vicinity of the limen insulae. The surgeon must be mindful of the possible proximity of the lateral lenticulostriate arteries, which may be damaged with manipulation of the distal M1 or proximal M2, and by carrying a resection deep to the anterior insula.

### 1.4. Surgical Outcomes

As insular resections for seizure control are relatively rare procedures, available data on postoperative seizure outcomes are limited to retrospective case series with relatively small numbers. The available data, however, indicate that rates of postoperative seizure freedom are favorable—typically in the range of 65–80%—and roughly comparable to resections for temporal lobe epilepsy [12]. Postoperative hemiparesis or worsening of existing motor deficits is common—as high as 40%—but this deficit is most commonly transient [7,8,12].

A recent meta-analysis by Obaid et al. found that, among 312 patients who underwent either open insular resection, LiTT, or radiofrequency ablation published in 24 retrospective studies, 66.7% of patients were seizure-free with a median follow-up duration of 2.58 years [7]. For patients who underwent LiTT or radiofrequency ablation, the seizure freedom rate was 50.7%, whereas, for open resection, the seizure freedom rate was 71.5%. Transient motor deficits were common following insular resection, occurring in 29.9% of cases. Most of these deficits were temporary, as permanent deficits were only present in 5% of cases. In this analysis, resection of the frontal operculum was independently associated with an increased risk of motor deficit, presumably due to damage to medullary arteries supplying the corona radiata or direct injury to the motor cortex or underlying white matter tracts [7]. There were no permanent language deficits.

In a recent study by Kudr et al., five out of thirty patients (16.7%) experienced hemiparesis due to corona radiata infarcts, four of which resulted in permanent deficits. All of these patients underwent resection of the posterior insula or the junction of the anterior and posterior insula [18]. A study by Bouthellier et al., however, found that frontal opercular resection and parietal opercular resection were each associated with corona radiata infarcts, suggesting that corona radiata lesions are not exclusive to posterior insular resection. Interestingly, this same study did not find that corona radiata ischemic lesions were independent predictors of motor deficits, and they, therefore, argued that motor deficits, in this case, are multifactorial, including possible contributions from ischemic injury to subcortical lesions, loss of function stemming directly from opercular resection, and retraction injury on the opercula. Furthermore, they argued that transient hemiparesis may be a consequence of injury to the insula itself, which has roles in motor function [19].

A further challenge in surgical decision-making stems from the distinction between lesional and non-lesional insular epilepsies. In epilepsy surgery, generally, epilepsy with an identifiable radiographic lesion is associated with superior outcomes. In insular epilepsy, many authors have argued against surgical intervention for patients with insular-onset epilepsy without a clear lesion [12]. Others have argued that palliative intervention with RNS may offer a superior risk–benefit profile in this patient population, though it is worth noting that in this series, although the majority of patients exhibited a reduction in seizure burden, none achieved seizure freedom [20]. With increasing familiarity with insular surgeries in the sEEG era, resection has become an increasingly common intervention for non-lesional insular epilepsies, and a recent meta-analysis found that 67.1% of cases exhibit Engel class I outcomes [21].

## 2. Institutional Case Series

A retrospective chart review was conducted of all patients from May 2017 to December 2024 who underwent open insular resection for drug-resistant epilepsy at the Children’s Hospital of Philadelphia. All surgeries were performed by the senior author (B.C.K.). Approval for this study was exempt from IRB review, per institutional IRB policy. For all patients, their parents provided consent for the procedures and for retrospective chart review research participation. Demographics, outcomes, and imaging data were obtained from the electronic medical record.

### 2.1. Case 1—Trans-Sylvian Approach for Insular Focal Cortical Dysplasia

A 9-year-old boy presented with a variable seizure semiology typically consisting of a facial pout followed by bilateral leg stiffening, left arm extension, and clonic movements that sometimes generalized to bilateral tonic–clonic seizures. These occurred roughly once per week. During scalp EEG evaluation, 20 seizures were captured in a 24 h period. He had mild left-sided motor weakness at baseline, which worsened in the post-ictal period. SEEG captured seizures with posterior insular onset, and open right insulectomy was recommended.

**Figure 1:**
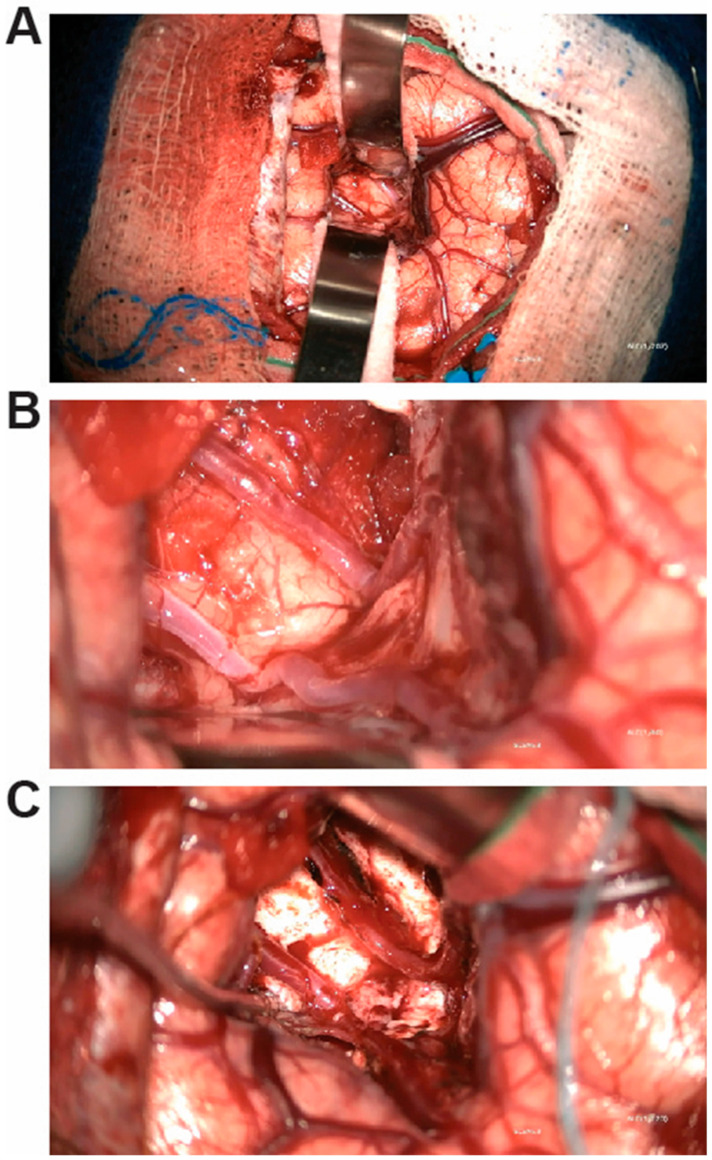
Intraoperative photographs of insular exposure and resection for Case 1. (**A**) Surgical exposure of posterior insula using trans-sylvian approach. (**B**) Photograph of insula to be resected along with overlying MCA branches. (**C**) Photograph following insular resection with preservation of MCA candelabra.

**Figure 2:**
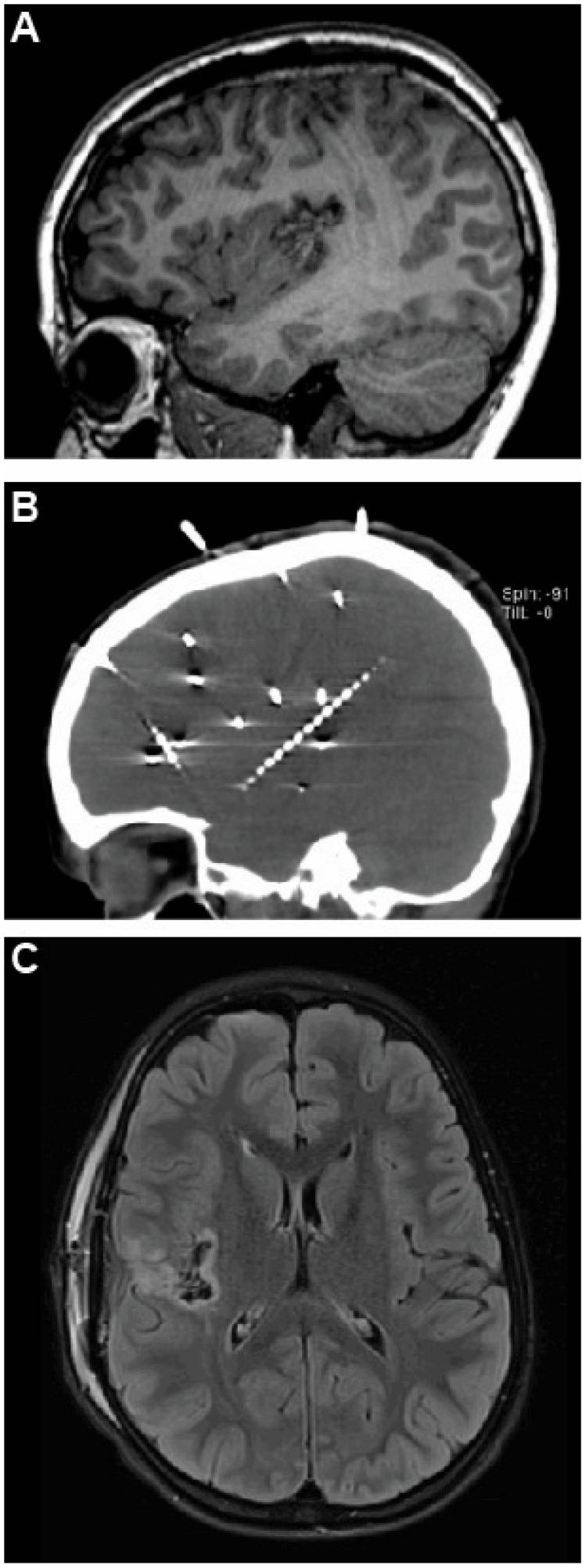
Postoperative sagittal T1-weighted MRI for Case 1 showing resection cavity (**A**), with prior sEEG CT scan for comparison (**B**). Postoperative FLAIR MRI showing posterior insular resection without corona radiata infarct (**C**).

At surgery, the Sylvian fissure was split posteriorly, exposing the aspects of the posterior insula implicated by sEEG (Figure 1A,B). MCA vessels were left in place, pia was cauterized and opened at the crown of each of three exposed gyri, and the cortex was resected down to white matter and along the insular sulci (Figure 1C). Surgical pathology was consistent with focal cortical dysplasia type IIA. There were no intraoperative complications, and there was no corona radiata infarct on postoperative MRI (Figure 2).

He was discharged and underwent outpatient physical therapy twice weekly and occupational therapy once weekly. As of three years follow-up, he was experiencing one seizure every 2–3 months of his usual semiology, all self-limited, his preoperative hemiparesis had resolved, and he was doing well in school and playing basketball.

### 2.2. Case 2—Trans-Opercular Resection for Focal Cortical Dysplasia in the Left Superior Temporal Gyrus and Posterior Insula

**Figure 3:**
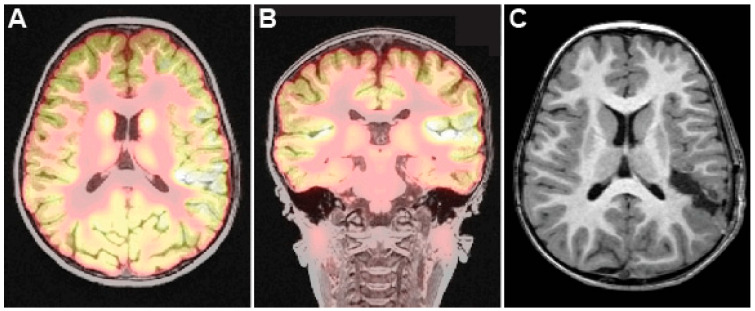
Pre- and postoperative imaging for Case 2. Axial (**A**) and coronal (**B**) preoperative PET-MRI demonstrating hypermetabolism in left posterior peri-sylvian region. (**C**) Postoperative axial T1-weighted MRI demonstrating resection of posterior insula and overlying opercula.

A 3-year-old girl with a history of focal seizures with impaired awareness and developmental/epileptic encephalopathy with spike-wave action in sleep (DEE-SWAS) presented for surgical evaluation. She experienced an explosive onset of epilepsy 9 months previously in the setting of COVID-19 infection. She experienced up to 100 daily seizures with semiology consisting of eye fluttering, staring, and occasionally left-hand automatisms. Scalp EEG and MEG revealed left temporal/temporoparietal-onset seizures. MRI-PET revealed hypermetabolism and a suspected region of cortical dysplasia involving the left perisylvian region, including the posterior insula (Figure 3A,B). While hypometabolism is typically associated with focal cortical dysplasia, regions of hypermetabolism common in DEE-SWAS correlate strongly with EEG hotspots [22,23]. Consistent with this, sEEG found frequent interictal spike-and-wave discharges in the posterior superior temporal gyrus and insula. SEEG captured 43 electroclinical seizures arising from the posterior insula and posterior superior temporal gyrus (STG). Given the concordant electro-anatamo-clinical findings, and in light of emerging evidence for the efficacy of surgery for improving outcomes of patients with DEE-SWAS [24,25], open resection of the seizure focus was recommended.

In surgery, the epileptogenic region of the posterior STG was circumferentially dissected and resected, with care taken to preserve the overlying vein of Labbé. The superior temporal sulcus, which represented the inferior extent of the superficial dissection, was skeletonized to its depth. The Sylvian fissure on the temporal side was skeletonized to its depth, allowing access to the posterior insula. All insular cortex implicated on SEEG was resected in a subpial fashion. Neuromonitoring signals remained stable throughout the case. There were no intraoperative complications, and there was no corona radiata infarct on postoperative MRI (Figure 3C).

Postoperatively, the patient exhibited no new neurological deficits, and she was noted to be increasingly communicative relative to baseline. She was admitted to inpatient rehabilitation for impaired coordination, impaired balance, and gait and functional mobility deficits, and she then underwent once-to-twice weekly outpatient physical therapy. Pathology revealed focal cortical dysplasia type 1A. She remains seizure-free (Engel class 1A) at three-years follow-up with improved cognitive and language function.

### 2.3. Case 3—Trans-Opercular Approach for Infarct Spanning Left Posterior Insula and Parietal Lobe

**Figure 4:**
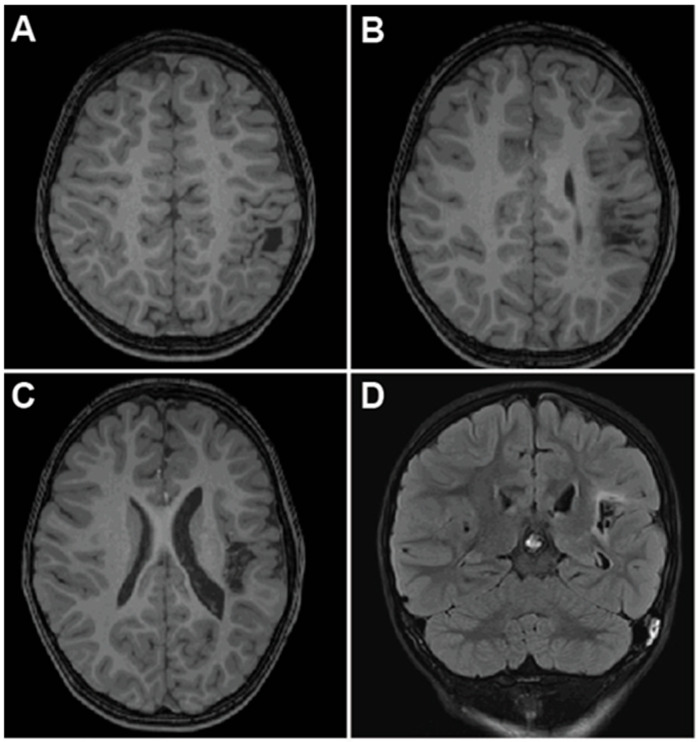
Preoperative axial T1-weighted (**A**–**C**) and coronal FLAIR (**D**) MRI for Case 3 demonstrating region of encephalomalacia spanning posterior insula through to the left parietal lobe.

A 5-year-old boy with a history of congenital heart disease necessitating a heart transplant developed a small left MCA stroke while on extracorporeal membrane oxygenation (ECMO). The stroke territory spanned an oblique course from the left parietal lobe, through the central lobule, to the posterior insula, and with some involvement of the STG (Figure 4). Among three seizure types (including one arising contralaterally), the patient’s most debilitating seizures were left-lateralized, occurring out of sleep, associated with rightward eye deviation and arm and leg twitching, with moaning and drooling typically lasting 25–40 min. Given his need for future cardiac MRIs, VNS and DBS were deemed suboptimal strategies. Open palliative resection was pursued. Preservation of motor function was of chief concern, given the lack of preoperative motor deficits.

At surgery, the exposed motor and sensory strips were mapped with monopolar stimulation. The gliotic aspects of the parietal and central regions were resected using ultrasonic aspiration. Resection was followed to the posterior insula, creating a surgical corridor without the need for additional resection or splitting of the fissure. The gray matter of the gliotic insula was resected using ultrasonic aspiration, with preservation of the white matter and all MCA branches. The boundaries of the area to be resected within the insula were determined based on anatomic and vascular landmarks correlated with navigation and preoperative imaging. Great care was taken to preserve all sulcal boundaries surrounding the resection bed and overlying MCA branches and to avoid invasion of the insular white matter.

**Figure 5:**
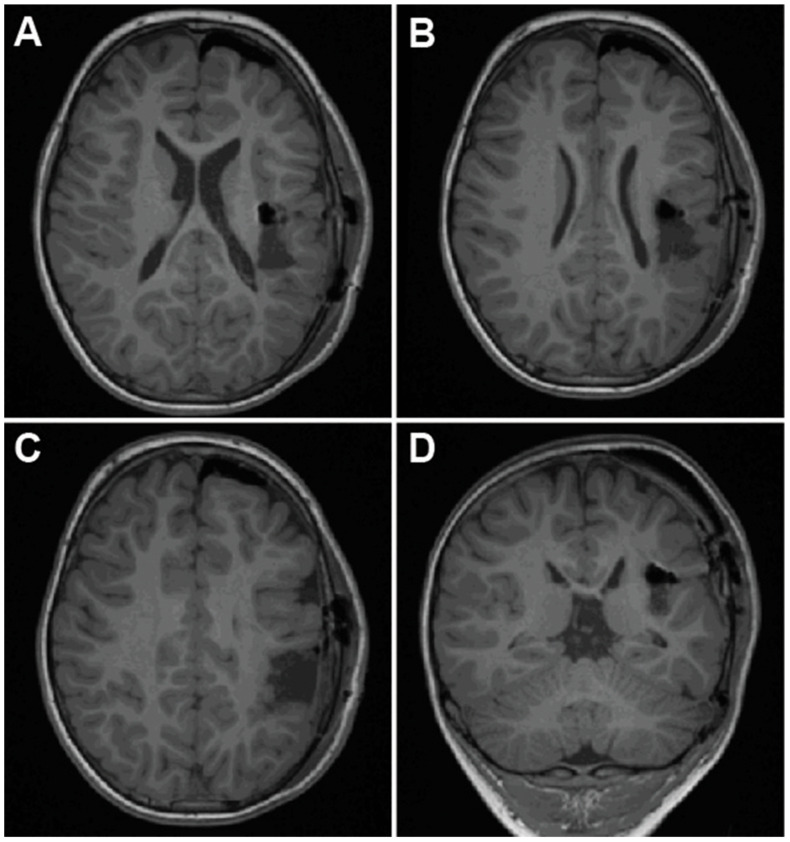
Axial (**A**–**C**) and coronal (**D**) postoperative T1-weighted MRI for Case 3 demonstrating resection cavity.

Postoperatively, the patient exhibited mild right arm weakness and reduced tone, particularly in the right wrist and finger extensors. He pursued outpatient physical and occupational therapy. On postoperative MRI, there was no evidence of corona radiata or internal capsule infarcts (Figure 5). Per family, his speech and diction have improved relative to before surgery. As of the 8-month follow-up, he has had two total seizures, one in the setting of illness and one following a missed medication. His postoperative motor deficit has resolved, although he has mild right-sided neglect, most noticeably in the right hand.

### 2.4. Case 4—Trans-Sylvian Approach for Idiopathic Insular Epilepsy

**Figure 6:**
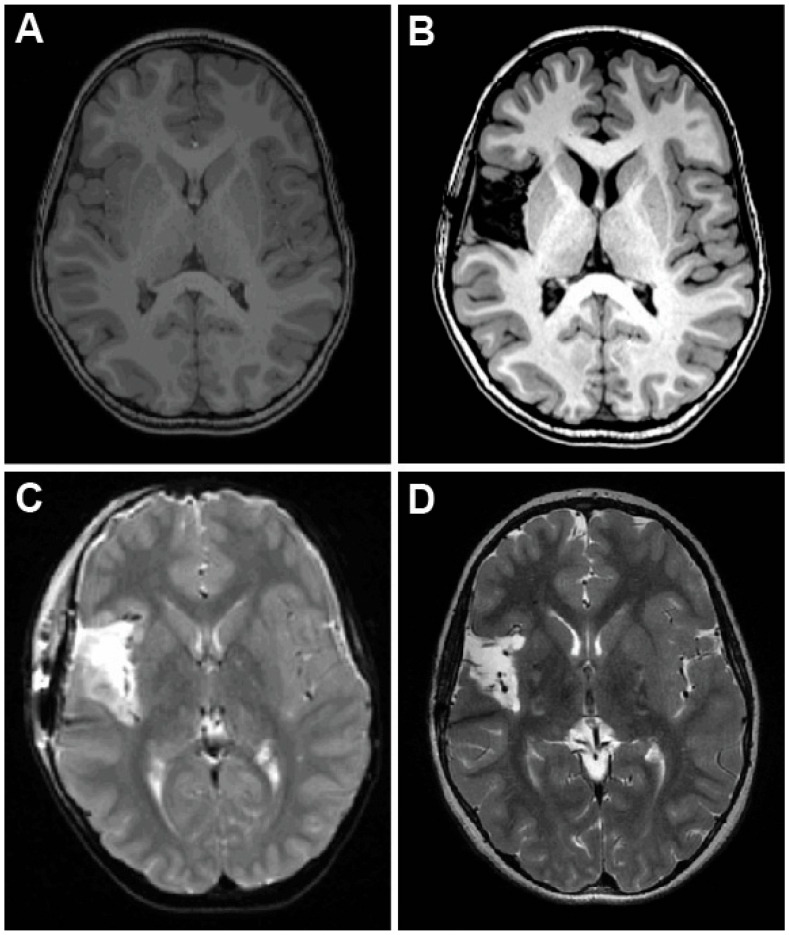
Pre- and postoperative MRI for Case 4 demonstrating complete insular resection. (**A**) Preoperative T1-weighted axial MRI demonstrating no focal anatomic abnormality in the right insular region. (**B**) Postoperative axial T1-weighted MRI demonstrating near-complete insulectomy. (**C**) Immediate postoperative axial T2-weighted MRI demonstrating cytotoxic edema in corona radiata and adjacent perisylvian frontal and temporal lobes, which resolved by 2 months postoperative MRI (**D**).

A 5-year-old boy with speech delay presented for surgical evaluation with a five-month history of multiple daily focal seizures consisting of head and arm paresthesias, left arm shaking, and garbled speech. He also experienced seizure clusters requiring rescue medications roughly once per three weeks. MRI was negative for a focal lesion (Figure 6A). PET revealed questionable hypometabolism within the right mesial temporal lobe, and MEG localized rare sharp activity to the right frontal lobe and anterior insula. Following sEEG implantation, 70 electroclinical seizures were captured with right insular onset, and a complete right insulectomy was recommended.

At surgery, the Sylvian fissure was split widely along its extent to expose the insula and circular sulcus. The resection of the insula began at the anterior extent of the circular sulcus near the limen insulae. The gray matter of the adjacent insula was dissected to the depth of the circular sulcus and carried circumferentially along the sulcus in a counterclockwise fashion. Once the circular sulcus was disconnected, the circumscribed insular gray matter was resected in a subpial fashion. Great care was taken to preserve all overlying blood vessels. Manipulation of MCA branches was minimized in order to reduce the risk of subsequent vasospasm. During resecting of the posterior insula, particular care was taken to preserve arterial perforators to minimize the risk of corona radiata infarct. A small piece of parietal operculum, which had been identified on sEEG, was also resected at this time. Next, the MCA was followed proximally to reach and resect the limen insulae. The superior amygdala was then disconnected through to the media pia of the uncus. Surgical pathology did not reveal any evidence of cortical dysplasia.

In the immediate postoperative period, he was noted to have a left-sided facial droop and mild left upper extremity weakness, and he was, therefore, recommended for outpatient physical and occupational therapy. Consistent with this, postoperative MRI revealed near-complete insulectomy, as dictated by the sEEG findings (Figure 6B) and small areas of cytotoxic edema in the corona radiata (Figure 6C). This weakness was resolved completely by the 2-month follow-up, and the cytotoxic edema resolved on subsequent MRI (Figure 6D). He was seizure-free for forty days postoperatively before seizures recurred, with repeat sEEG revealing broad electrographic onset in the superficial face motor, deep premotor, deep mouth motor, and deep opercular areas. The patient underwent RNS placement with temporary improvement of seizure burden but subsequently developed atonic and tonic drop seizures consistent with Lennox-Gastaut syndrome. By 18 months post-insulectomy, he was experiencing upwards of 20 seizures daily and as many as 100, primarily drop seizures. Given his high burden of drop attacks, he underwent corpus callosotomy, which resolved all drop attacks and generalized seizures. As of the two-year follow-up, he experiences seven to eight daily focal seizures with preserved awareness, although he can experience several days of seizure-free periods.

## 3. Summary

Of these four patients, three exhibited postoperative motor deficits. Motor deficits have resolved in all patients, excluding ongoing mild right-sided neglect in one patient. One patient has experienced a postoperative motor improvement with resolution of their preoperative motor deficits. No patients suffered intraoperative infarcts affecting the corona radiata or any other aspect of the corticospinal tracts. The cases described herein represent both the challenges of insular resection and the technical nuances that assist in minimizing adverse motor outcomes.

## 4. Conclusions

The insula is increasingly recognized as an important yet insidious source of focal, drug-resistant epilepsy. Both LiTT and open resection represent viable treatment options for insular seizures, though open resection is associated with superior postoperative seizure control. Posterior insular surgeries are frequently associated with transient hemiparesis. Safe posterior insular surgery is contingent upon a thorough understanding of insular anatomy and careful surgical technique. Care must be exercised to preserve deep insular arteries and medullary arteries supplying the corona radiata, which requires thorough knowledge of the anatomy.

## Data Availability

The original contributions presented in this study are included in the article. Further inquiries can be directed to the corresponding author.
